# Management of acute myocardial infarction in chronic kidney disease in Germany: an observational study

**DOI:** 10.1186/s12882-025-03943-5

**Published:** 2025-01-09

**Authors:** Victor Walendy, Andreas Stang, Matthias Girndt

**Affiliations:** 1 Department of Internal Medicine II, Universitätsmedizin (Halle), Medical Faculty of the Martin-Luther-University Halle-Wittenberg, Ernst-Grube-Straße 40, 06120 Halle (Saale), Germany; 2 Zentrum für Klinische Epidemiologie, Universitätsklinikum Essen, Universität Duisburg- Essen, Essen, Germany; 3 Department of Epidemiology, School of Public Health, Boston University, Boston, MA USA

**Keywords:** Myocardial infarction, Chronic kidney disease, Dialysis, Contrast-associated acute kidney injury, Coronary intervention

## Abstract

**Background:**

Managing acute myocardial infarction (AMI) in patients with chronic kidney disease (CKD) or end-stage renal disease on dialysis (renal replacement therapy, RRT) presents challenges due to elevated complication risks. Concerns about contrast-related kidney damage may lead to the omission of guideline-directed therapies like percutaneous coronary intervention (PCI) or coronary artery bypass grafting (CABG) in this population.

**Methods:**

We analysed German-DRG data of 2016 provided by the German Federal Bureau of Statistics (DESTATIS). We included cases with a primary diagnosis of AMI (ST-Elevation Myocardial Infarction (STEMI) or Non-ST-Elevation Myocardial Infarction (NSTEMI) ICD-10: I21 or I22) with and without CKD or RRT. We calculated crude- and age-standardized hospitalization rates (ASR, per 100,000 person years). Furthermore, we calculated log-binominal regression models adjusting for sex, CKD, RRT, comorbidities, and place of residence to estimate adjusted relative-risks (aRR) for receiving treatments of interest in AMI, such as PCI or CABG.

**Results:**

We identified 217,514 AMI-cases (69,728 STEMI-cases and 147,786 NSTEMI-cases). AMI-cases without CKD had percutaneous coronary intervention (PCI) in 60.8%. In contrast, AMI-cases with CKD or RRT had PCI in 46.6% and 54.5%, respectively. The ASR for AMI-cases amounted to 184.7 (95%CI 183.5-185.8) per 100,000 person years. In regression analysis AMI-cases with CKD were less likely treated with PCI (aRR: 0.89 (95%CI 0.88–0.90)), compared to cases without CKD. AMI-Cases with RRT showed no difference in PCI rates (aRR: 1.0 (95%CI 0.97–1.03)) but were more frequently treated with CABG (aRR: 2.20 (95%CI 2.03–2.39)). Conversely, CKD was negatively associated with CABG (aRR: 0.71, 95%CI 0.67–0.75) when non-CKD cases were used as the reference group.

**Conclusion:**

We show that AMI-cases with CKD underwent PCI less frequently, while RRT has no discernible impact on PCI utilization in AMI. Furthermore, AMI-cases with RRT exhibited a higher CABG rate.

**Supplementary Information:**

The online version contains supplementary material available at 10.1186/s12882-025-03943-5.

## Key learning points

**What was known:**

• In 2004, Glenn Chertow et al. coined “renalism” for low coronary intervention rates in chronic kidney disease. Fears of contrast-associated acute kidney injury led to avoiding invasive diagnostics in CKD patients with myocardial infarction. Mostly North American cohort studies consistently link underuse of percutaneous coronary intervention to higher mortality and morbidity in CKD patients.

• Since then, awareness for this disadvantage has heightened and precise guidelines for the prevention of contrast-associated renal complications have been published.

**This study adds:**

• This nationwide study explores healthcare for patients with chronic kidney disease (CKD) and acute myocardial infarction.

• Findings show consistently lower percutaneous coronary intervention (PCI) rates with CKD. Remarkably, cases with renal replacement therapy (RRT) had PCI rates similar to those without CKD.

• “Renalism” remains a concern in Germany’s healthcare landscape.

**Potential impact:**

• The outcomes of our study underscore the need for a thorough re-evaluation of the treatment approach for patients with chronic kidney disease (CKD) experiencing acute myocardial infarction.

• We propose that this data serves as a catalyst for raising awareness and initiating campaigns aimed at mitigating the impact of “renalism” in routine patient care.

**Supplementary Information:**

The online version contains supplementary material available at 10.1186/s12882-025-03943-5.

## Introduction

Acute myocardial infarction (AMI) is a life-threatening condition that requires prompt and appropriate medical intervention. The management of AMI in patients with chronic kidney disease (CKD) or end-stage renal disease (ESRD) with dialysis dependency (renal replacement therapy, RRT) poses substantial challenges due to the increased risk of complications and concerns regarding adverse events associated with the use of contrast media in these patients [1–3]. While contrast-associated acute kidney injury (CA-AKI) is thought to promote progressive kidney dysfunction, a causal relationship between contrast use and increases in serum creatinine thereafter remains unproven [4–6]. Evidence mounts that clinically indicated, potentially lifesaving radiographic procedures are underutilized in patients with CKD [7–9]. European Guidelines acknowledge the underuse of myocardial revascularization of CKD patients and recommend a similar diagnostic or interventional approach as in non-CKD patients [10–12]. Nevertheless, recent studies have revealed a persistent trend of underutilization of coronary angiography (CA) or PCI in patients with CKD or ESRD [7–9, 12, 13]. The purpose of this study is to investigate differences in the care of CKD patients with AMI compared with patients without CKD in Germany.

## Material & methods

We used German diagnosis-related group (DRG) data provided by the German Federal Bureau of Statistics (DESTATIS). According to § 21 KHEntgG (Hospital Charges Act), all hospitals must submit their hospitalization data annually to the Hospital Remuneration System (InEK) covering virtually all hospitals in Germany to be renumerated. The data is finally anonymized and forwarded to DESTATIS for further scientific use. This data can be analysed using remote data processing and contain information on age at admission, sex, length of stay (LOS), location of residence by federal state and of the treating hospital of each hospitalization. Moreover, up to eighty-nine secondary and one primary diagnosis in the International Classification of Diseases, 10th edition (ICD-10) format are embedded in the DRG data. Up to one hundred procedures and interventions may be coded using the current “Operationen- and Prozedurenschlüssel (OPS)” classification system. We analysed hospitalizations of the year 2016. The structure of DRG-data supplied by the Federal Bureau of Statistics has been described in detail elsewhere [14, 15]. Further information is available online (https://www.forschungsdatenzentrum.de/en/health/drg).

We analyzed hospitalizations with a primary diagnosis of an acute myocardial infarction (ST-Elevation Myocardial Infarction (STEMI) or Non-ST-Elevation Myocardial Infarction (NSTEMI): ICD-10 I21 or I22). To determine the treatment modality, we searched for angioplasty codes (OPS: 8-837.0) or OPS-Codes indicating CABG (OPS: 5-36x). When PCI was used for revascularization, we further assessed whether a drug-eluting stent or balloon (DES/DEB, OPS: 8-837.m, 8-837.v, 8-837.w) or a bare-metal stent was implanted (BMS, OPS: 8-837.k, 8-837.u). If no intervention occurred, we assumed coronary angiography (CA). We identified hospitalizations in patients with chronic kidney disease stages 3 to 5 (CKD) by their respective ICD Codes (N18.3-5) representing the actual stage of kidney disease. Furthermore, renal replacement therapy (RRT) was assumed if one of the common OPS or ICD codes were present. A detailed list of ICD or OPS codes is provided in supplementary Table 1 (Supplement Table S1). We used a modified Charlson-Comorbidity-Index (mCCI) [16], which contains only comorbidities of chronic nature, to assess for comorbidities. We removed ICD-10-Codes for AMI from the mCCI (ICD-10: I21, I22). The DRG-Data is anonymized, readmissions could contribute to this dataset more than once.

We excluded hospitalizations because of missing data on sex (*n* = 3) or unknown place of residence (*n* = 1,666).

### Statistical methods

The unit of analysis was the hospital admission with a primary diagnosis of an acute myocardial infarction (AMI). We defined three subgroups: AMI cases without CKD, AMI cases with CKD but not ESRD, and ESRD cases with RRT. We calculated crude- and age-specific rates (per 100,000 person years) for the different treatment modalities separately by sex. In addition, we performed direct age-standardization using the new European Standard Population [17]. We calculated exact confidence intervals (CI) of the rates [18]. Furthermore, we calculated log-binominal regression models to estimate adjusted relative-risks (aRR) for receiving treatments of interest after AMI, such as PCI or CABG. To estimate differences in the length of stay, depending on the different covariates, we performed a Poisson regression to calculate the respective rate ratios (RR). For each model minimal sufficient adjustment sets were generated with the use of directed acyclic graphs [19]. All models were adjusted for place of residence (federal state). Statistical analysis was performed using SAS 9.4 (Statistical Analysis Software 9.4, SAS Institute Inc, Cary, North Carolina, USA).

## Results

We identified in total 217,514 AMI-cases in Germany in 2016, including 69,728 STEMI-cases and 147,786 NSTEMI-cases. There were 6,209 (9.9%) STEMI-cases with CKD and 850 (1.4%) with RRT. Further, there were 26,772 (22.6%) NSTEMI-cases with CKD and 2,680 (2.3%) with RRT, respectively (Table 1). The overall age-standardized hospitalization rate (ASR) for STEMI-cases amounted to 62.3 (95%CI 61.8–62.8) per 100,000 person years, whereas the ASR for NSTEMI-cases was 122.4 (95%CI 121.7–123.0) per 100,000 person years.

**Table 1:** German-wide hospitalizations for acute myocardial infarctions in Germany, 2016 by type of acute myocardial infarction and kidney disease status

	STEMI			NSTEMI		
	CKD	RRT	Non-CKD	CKD	RRT	Non-CKD
Hospitalizations (n)	6,209	850	62,669	26,772	2,680	118,334
Males (%)	56.3	70.7	71.1	58.2	70.2	66.3
Crude rate (per 100.000 person years)	7.7	1.0	67.4	33.2	3.3	107.2
Age-standardized rate (per 100.000 person years), 95% CI	4.8	0.7	56.8	19.8	2.2	100.4
upper CI	4.7	0.7	56.3	19.6	2.1	100.0
lower CI	4.9	0.8	57.3	20.0	2.3	100.8
Age (years, median, P10, P90)						
Male	76 (59, 87)	68 (52,82)	63 (48, 80)	79 (65, 88)	74 (57, 84)	71 (52, 85)
Female	81 (67, 91)	75 (54,86)	74 (52, 87)	82 (70, 91)	76 (59, 84)	78 (58, 89)
Total	78 (62, 89)	70 (53, 84)	65 (49, 83)	80 (67, 89)	75 (58, 84)	74 (53, 87)
Length of stay (days, median, P10, P90)	8 (2, 22)	17 (3, 49)	6 (1, 16)	8 (2, 21)	12 (3, 37)	6 (1, 16)
In-hospital mortality (n, %)						
Male	556 (15.9)	207 (34.4)	4,759 (10.7)	1,421 (9.1)	394 (20.9)	5,686 (7.2)
Female	501 (18.4)	106 (42.6)	3,168 (17.5)	1,091 (9.7)	175 (21.9)	4,127 (10.4)
Total	1,057 (17.0)	313 (36.8)	7,927 (12.7)	2,512 (9.4)	569 (21.2)	9,813 (8.3)
CKD-Stages (n, %)						
Stage 3	4,862 (78.4)			20,400 (76.0)		
Stage 4	1,054 (17.0)			4,996 (18.6)		
Stage 5 (non-dialysis)	287 (4.6)			1,458 (5.4)		
Comorbidities (mCCI) (median, P10, P90)	2 (0, 4)	2 (1, 5)	1 (0, 3)	2 (0, 4)	3 (1, 6)	1 (0, 3)
Coronary heart disease (CHD) (n, %)						
Three-vessel	2,758 (44.4)	452 (53.2)	20,876 (33.3)	12,991 (48.5)	1,706 (63.7)	45,814 (38.7)
Two-vessel	1,403 (22.6)	189 (22.2)	16,293 (26.0)	4,739 (17.7)	437 (16.3)	24,495 (20.7)
One-vessel	969 (15.6)	102 (12.0)	16,921 (27.0)	2,838 (10.6)	220 (8.2)	19,525 (16,5)
NA	1.079 (17,4)	107 (12,6)	8.579 (13,7)	6.204 (23,2)	317 (11,8)	28.497 (24,1)

STEMI: ST-Segment elevating myocardial infarction; NSTEMI: Non-ST-segment elevation myocardial infarction; CKD: Chronic kidney disease; RRT: Renal replacement therapy; PCI: Percutaneous coronary intervention; CABG: Coronary artery bypass graft; mCCI: modified Charlson Comorbidity Index; CHD 1–3: Coronary heart disease; CI: Confidence interval

### General characteristics

The observed median age differed between male and female cases and between CKD, RRT and those with non-CKD status (Table 1). STEMI-cases or NSTEMI-cases with CKD tended to be older than non-CKD patients (median age-difference STEMI: 13 years, NSTEMI: 6 years). This was less pronounced in cases with RRT (median age-difference STEMI: 5 years, NSTEMI: 1 year). In general, we observed more male AMI-cases. This persisted throughout the subgroups of CKD, RRT and non-CKD cases (Table 1). The median length of stay of STEMI-cases differed considerably between CKD (8 days (P10: 2, P90: 22)) and RRT (17 (P10: 3, P90: 49)) (Table 1). This was similar in NSTEMI-cases, hospitalizations with RRT stayed in median four days longer (12 days (P10: 3, P90: 37)) (Table 1) than those with CKD. In a Poisson regression the age- and sex-adjusted rate ratio (RR) for an increased LOS was 2.45 (95% CI 2.28–2.62) in STEMI-cases with RRT, meaning an almost two and a half times increased LOS compared to STEMI-cases without CKD. The median LOS of NSTEMI-cases with RRT was nearly twice as long compared to NSTEMI-cases without CKD (RR 1.89, 95%CI 0.82–0.97).

The overall in-hospital mortality for AMI-cases was 9.8% in non-CKD cases and 12.2% in CKD and 25.0% in RRT cases (STEMI 36.8% and NSTEMI 21.2%, Table 1).

The age-specific rates peaked in STEMI-cases and NSTEMI-cases with CKD for both sexes in the age group of 80 to 85 and above (Supplemental Fig. S1). In contrast, STEMI-cases and NSTEMI-cases with RRT showed a steep decline of age-specific rates in the age group of 85 years and above (Supplement Fig. S1 and S2). Female STEMI- or NSTEMI-cases with CKD showed a tableau or less steep increase in age-specific rates in the age group 85+ (Supplement Fig. S1). The age-specific rates of the non-CKD group behave similarly overall, only in the age group 70–74, there is a moderate decrease in the age-specific rates (Supplement Fig. S3).

### Management of acute myocardial infarction

#### STEMI

**Fig. 1:**
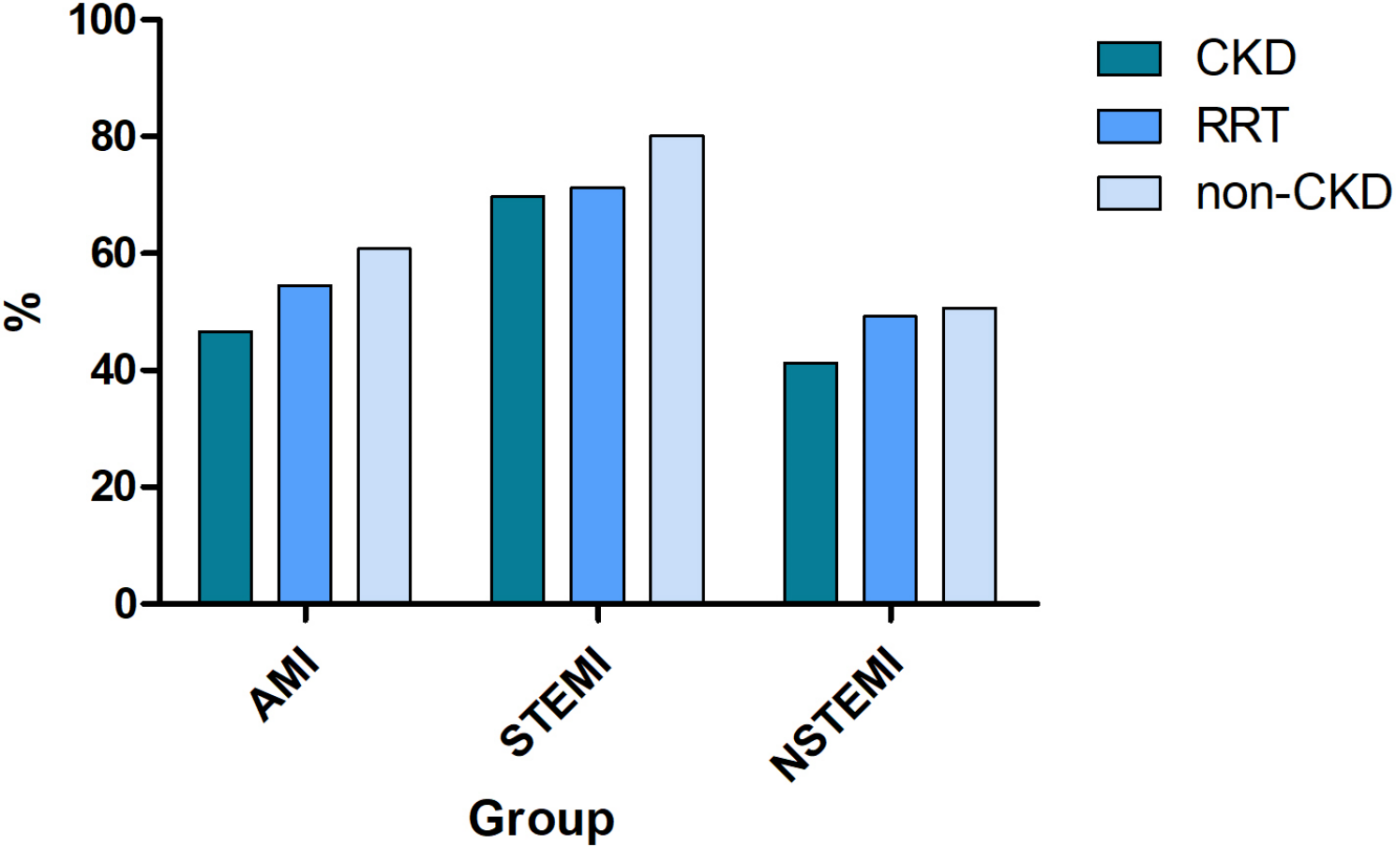
Relative frequency of PCI in AMI, STEMI and NSTEMI in Germany by CKD- and RRT-Status. STEMI: ST-Segment elevating myocardial infarction; NSTEMI: Non-ST-segment elevation myocardial infarction; CKD: Chronic kidney disease; RRT: Renal replacement therapy

STEMI-cases with CKD or RRT were treated with PCI only in 69.7% and 71.2%, respectively (Fig. 1). In contrast, STEMI- cases without CKD were treated by PCI in 80.1% (Fig. 1). Surgical revascularization (CABG) was used more frequently in RRT cases (16.7%) than in CKD (4.1%) or non-CKD- (3.5%) cases, respectively (Table 2). This difference was most pronounced in male RRT cases (Table 2). The relative frequency of conservative treatment was 9.2% points higher for STEMI-cases with CKD compared to non-CKD (Table 2). The binominal regression showed that STEMI-cases were less likely to receive PCI with increasing age, female sex, increase in mCCI, and the presence of CKD (Supplement Fig. S4). Interestingly, there was no clear association for hospitalizations with RRT (Supplement Fig. S4). The presence of CKD was associated with a lower probability (given as adjusted relative risk (aRR)) to be treated with PCI by 11% (aRR 0.89 (95%CI 0.88–0.90)). Similarly, female sex was associated with a lower probability to receive PCI in STEMI (aRR 0.89 (95%CI 0.88–0.90)) (Supplement Fig. S4).

**Table 2:** Treatment modalities of myocardial infarction in German hospitals 2016 by type of acute myocardial infarction and kidney disease status

	STEMI			NSTEMI		
PCI (n, % of all hospitalizations)	CKD	RRT	Non-CKD	CKD	RRT	Non-CKD
Male	2,542 (72.8)	422 (70.2)	36,511 (82.0)	6,916 (44.4)	950 (50.5)	42,616 (54.3)
Female	1,783 (65.7)	183 (73.5)	13,682 (75.5)	4,131 (36.9)	369 (46.2)	17,302 (43.4)
Total	4,325 (69.7)	605 (71.2)	50,193 (80.1)	11,047 (41.3)	1,319 (49.2)	59,918 (50.6)
Diagnostic coronary angiography (n, %)	389 (6.3)	67 (7.9)	3,312 (5.3)	5,076 (19.0)	563 (21.0)	23,498 (19.9)
CABG (n, %)						
Male	185 (5.3)	110 (18.3)	1,759 (3.9)	832 (5.3)	314 (16.7)	6,222 (7.9)
Female	68 (2.5)	32 (12.8)	465 (2.6)	319 (2.8)	122 (15.3)	1,673 (4.2)
Total	253 (4.1)	142 (16.7)	2,224 (3.5)	1,151 (4.3)	436 (16.3)	7,895 (6.7)
Conservative (n, %)						
Male	766 (21.9)	69 (11.5)	6,270 (14.1)	7,833 (50.3)	617 (32.8)	29,638 (37.8)
Female	865 (31.9)	34 (13.7)	3,982 (22.0)	6,741 (60.2)	308 (38.6)	20,883 (52.4)
Total	1,631 (26.3)	103 (12.1)	10,252 (16.4)	14,574 (54.4)	925 (34.5)	50,521 (42.7)

STEMI: ST-Segment elevating myocardial infarction; NSTEMI: Non-ST-segment elevation myocardial infarction; CKD: Chronic kidney disease; RRT: Renal replacement therapy; PCI: Percutaneous coronary intervention; CABG: Coronary artery bypass graft

In STEMI-cases treated with PCI, we observed comparable frequencies for CKD and non-CKD in the use of drug-eluting stents (DES), bare-metal stents and drug-eluting balloons (DEB). Only the use of BMS was slightly more frequent in CKD and RRT cases compared to non-CKD cases (6.5% vs. 3.3%). After adjusting for age, sex, and place of residence, we estimated increased aRR for undergoing CABG in STEMI-cases with RRT (aRR: 3.22 (95% 2.74–3.80)), whereas CKD had no measurable influence (aRR: 0.95 (95%CI 0.83–1.09)).

Diagnostic coronary angiography (CA) without revascularization was performed slightly more frequently in hospitalizations with RRT (7.9%) compared to non-CKD hospitalizations (5.3%) (Table 2).

#### NSTEMI

The primary therapeutic modality in NSTEMI was PCI regardless of the presence of chronic kidney disease (Table 2). The relative frequency of PCI was 9.3% lower in CKD- compared with non-CKD NSTEMI-cases (Fig. 1). There was virtually no difference in the use of PCI between RRT and non-CKD hospitalizations (Table 2). Surgical revascularization (CABG) was more common in the RRT group when compared to CKD and non-CKD NSTEMI-cases (Table 2). We observed the highest rates of conservative treatment in CKD (54.4%) compared to non-CKD NSTEMI-cases (42.7%), respectively. RRT NSTEMI-cases had the lowest rate of conservative treatment (34.5%). In multivariable analysis, we found a negative association to receive PCI after adjustment for sex, age, mCCI and place of residence when CKD was present (aRR 0.95 (95%CI 0.94–0.97)). RRT was even positively associated with PCI in this situation (Supplement Fig. S5). CABG after adjustment for the aforementioned covariates, was positively associated with RRT (aRR: 1.95 (95%CI 1.77–2.14)), in contrast to CKD (aRR: 0.66 (95%CI 0.61–0.70)).

#### AMI

**Fig. 2:**
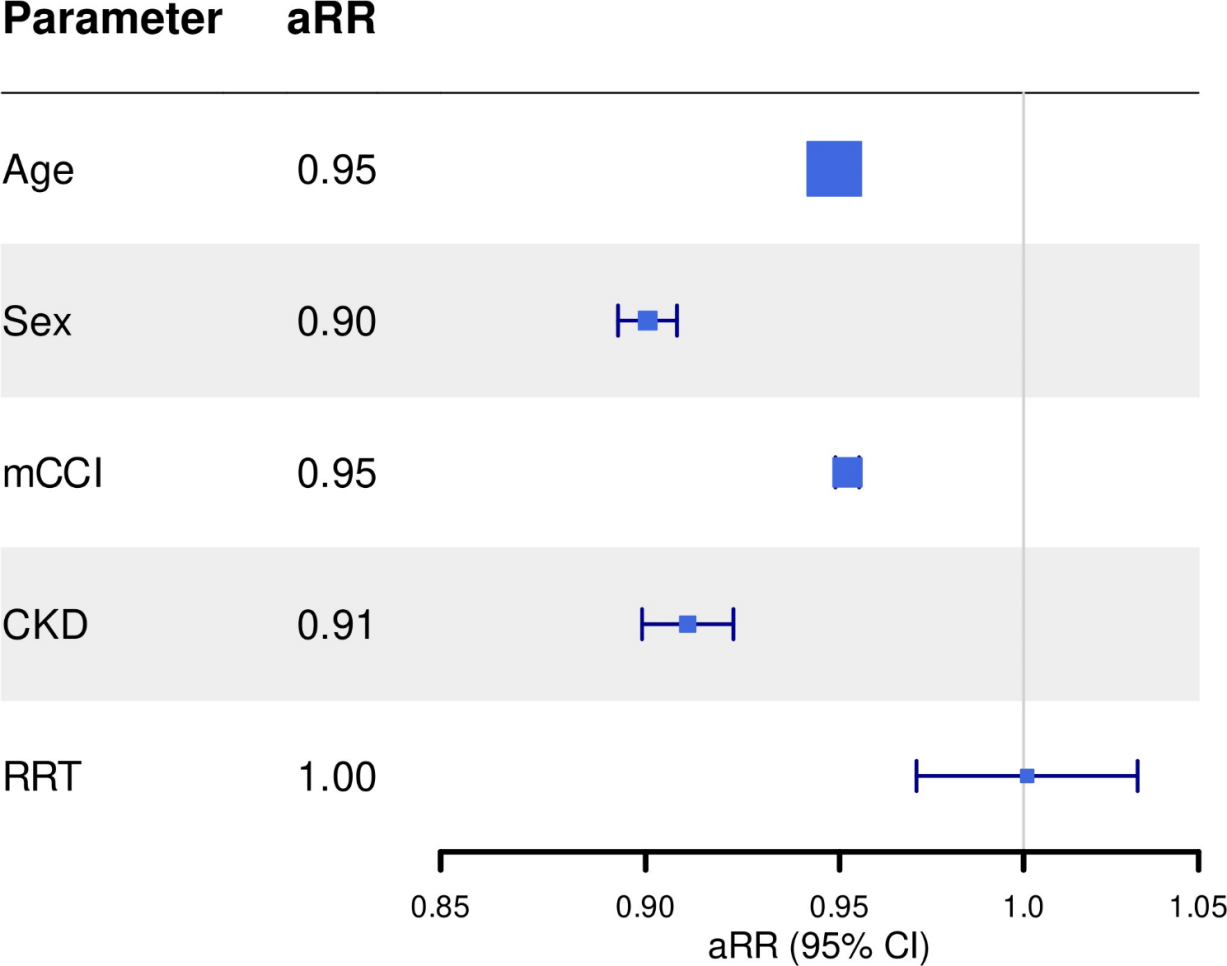
PCI in acute myocardial infarction in Germany. Plotted regression coefficients and 95% confidence Intervals. CKD: Chronic kidney disease; RRT: Renal replacement therapy; mCCI: modified Charlson Comorbidity Index

The overall treatment of acute myocardial infarction (STEMI and NSTEMI) in CKD AMI-cases differed distinctly from AMI-cases without renal dysfunction. CKD was negatively associated with the use of PCI (aRR: 0.89 (95%CI 0.88–0.90)), after adjustment for sex, age, mCCI and place of residence (Fig. 2). In contrast, treatment of AMI-cases with RRT did not clearly differ from the non-CKD population (Fig. 2). CABG was more often performed in RRT AMI-cases (aRR 2.20 (95%CI 2.03–2.39)). Whereas CKD was negatively associated with CABG (aRR: 0.71 (95%CI 0.67–0.75)).

## Discussion

Amongst 217,514 AMI-cases in Germany in 2016, we observed that those with CKD were less likely to receive PCI, regardless of the type of myocardial infarction (STEMI or NSTEMI). In our binominal regression analysis, covariates, which negatively affected the probability of receiving PCI, were age, female sex, the number of comorbidities and the presence of CKD. The relative frequency of conservative treatment in STEMI- or NSTEMI-cases was the highest in the CKD group.

In STEMI-cases RRT showed no effect on the probability of PCI treatment. In NSTEMI-cases RRT was positively associated with PCI treatment. The percentage of conservative treatment in this group was even lower compared to the non-CKD group. Regarding treatment modality, STEMI-cases with RRT had the highest rate of surgical revascularization. This was also evident for NSTEMI-cases with RRT. This finding is likely due to the higher complexity of coronary artery disease in this patient group [20] and current guideline recommendations [10, 11]. In STEMI-cases the median LOS was almost 2.5 times longer in the RRT group, in NSTEMI-cases with RRT the median LOS nearly doubled, compared to non-CKD STEMI-cases. The relative frequency of in-hospital mortality was expectedly the highest in STEMI- and NSTEMI-cases with RRT.

We showed a reduced probability of PCI treatment in AMI-cases with CKD. This finding coincides with an analysis of the National Inpatient Sample (NIS) of the US from 2017. The authors report that CKD patients were less likely to undergo PCI or CABG in AMI (adjusted OR 0.60, 95% CI 0.59–0.61) compared to non-CKD patients [3]. Like in our findings, cases with RRT had a higher rate of surgical revascularization and were more likely to be invasively managed compared to CKD cases. A recent publication of data from a national registry of North-New Zealand (ANAZCS-QI 70) included 23,432 cases between 2013 and 2018, of which approximately 40% had CKD. It was likewise observed that invasive reperfusion strategy (PCI) was used inversely proportional to increasing CKD stage. In this cohort, like in our findings, there was no difference in the utilization of PCI in RRT cases compared to non-CKD cases [21]. Kawsara et al. again queried the US-NIS-Data (2016–2018) and compared STEMI-Management in dialysis and non-dialysis cases [12]. The dialysis cases were less likely to undergo PCI (0.58 (95% CI, 0.50–0.68)), and they found no difference in the utilization of bypass surgery between the two groups [22]. An analysis of SWEDEHEART data (2011–2014) of patients with NSTEMI aged 80 years and above revealed that only 22% of CKD G3 and 10% of CKD G4-5 patients underwent PCI [23]. The authors performed a proportional hazard regression for in-hospital death comparing a non-PCI vs. a PCI strategy and observed a remarkable advantage for PCI (0.44 (0.33–0.59)), though confounding by indication could be an issue in such an analysis.

CKD has been traditionally associated with worse outcomes after PCI [24]. Additionally, the fear to induce CA-AKI might have further complicated treatment decisions. One might speculate that because of the need for radiocontrast administration, vital therapies, such as PCI, may be less frequently performed in patients with CKD to avoid CA-AKI. The so-called ”renalism” has been discussed for several decades now [25]. This may also explain our observation, in which RRT AMI-cases were equally likely to undergo PCI compared to non-CKD AMI-cases. CKD and RRT cases were markedly older and had more comorbidities compared to non-CKD cases.

Restricting access for CKD patients to important therapeutic interventions such as PCI in AMI, although there are guideline recommendations and data suggesting a treatment benefit, is a clear disparity in the management of AMI in CKD patients [3, 10, 11, 26]. This should be addressed when conducting awareness campaigns.

We showed high rates of CABG treatment in AMI-cases with RRT. This practice reflects observational evidence, in which long-term survival was improved after CABG compared to PCI in this cohort [10, 11, 27]. It must be stressed that there are no controlled randomized trials on this question.

### Strengths and limitations

One of the major strengths of this study is the large sample size drawn from virtually all hospitals in Germany. This is a unique opportunity to study the clinical care of CKD or RRT hospitalizations. Until now, there is no population-based data on the in-hospital management of AMI in CKD or RRT hospitalizations in Germany.

This study has limitations, which emerge from the observational study design. This data is more susceptible to undetected confounding, which we could not adjust for, as clinical parameters are lacking (e.g. severity of MI, hemodynamic stability, obesity, frailty, or patient preferences). Furthermore, in regression analysis non-linear trends could have led to bias, as we assumed linear interaction in our models. Finally, healthcare data is only as good as the coding quality in the respective hospitals.

## Conclusion

AMI-Cases with CKD were less invasively treated compared to non-CKD cases even after adjustment for confounders. This finding is in line with other studies on the topic and could reflect a so called “renalism” in this population, i.e. reluctance to use optimal therapeutic strategies due to fear of side-effects. In-hospital mortality was high for CKD and RRT hospitalizations, especially for hospitalizations with STEMI and CKD or RRT. Hospitalizations with RRT had a higher rate of surgical revascularization (CABG), which reflects current guideline recommendations.

## Electronic supplementary material

Below is the link to the electronic supplementary material.

Supplementary Material 1

Supplementary Material 2

Supplementary Material 3

Supplementary Material 4

Supplementary Material 5

Supplementary Material 6

## Data Availability

The data that support the findings of this study are openly available in figshare at http://doi.org/10.6084/m9.figshare.24759798.
