# Supplementary material for: Management of acute myocardial infarction in chronic kidney disease in Germany: an observational study
Source: BMC Nephrol. 2025 Jan 9;26:15. doi: 10.1186/s12882-025-03943-5 (PMC11720599; doi:10.1186/s12882-025-03943-5)
Supplement: Supplementary file 4 — Supplementary Material 4 [file 12882_2025_3943_MOESM4_ESM.pdf]

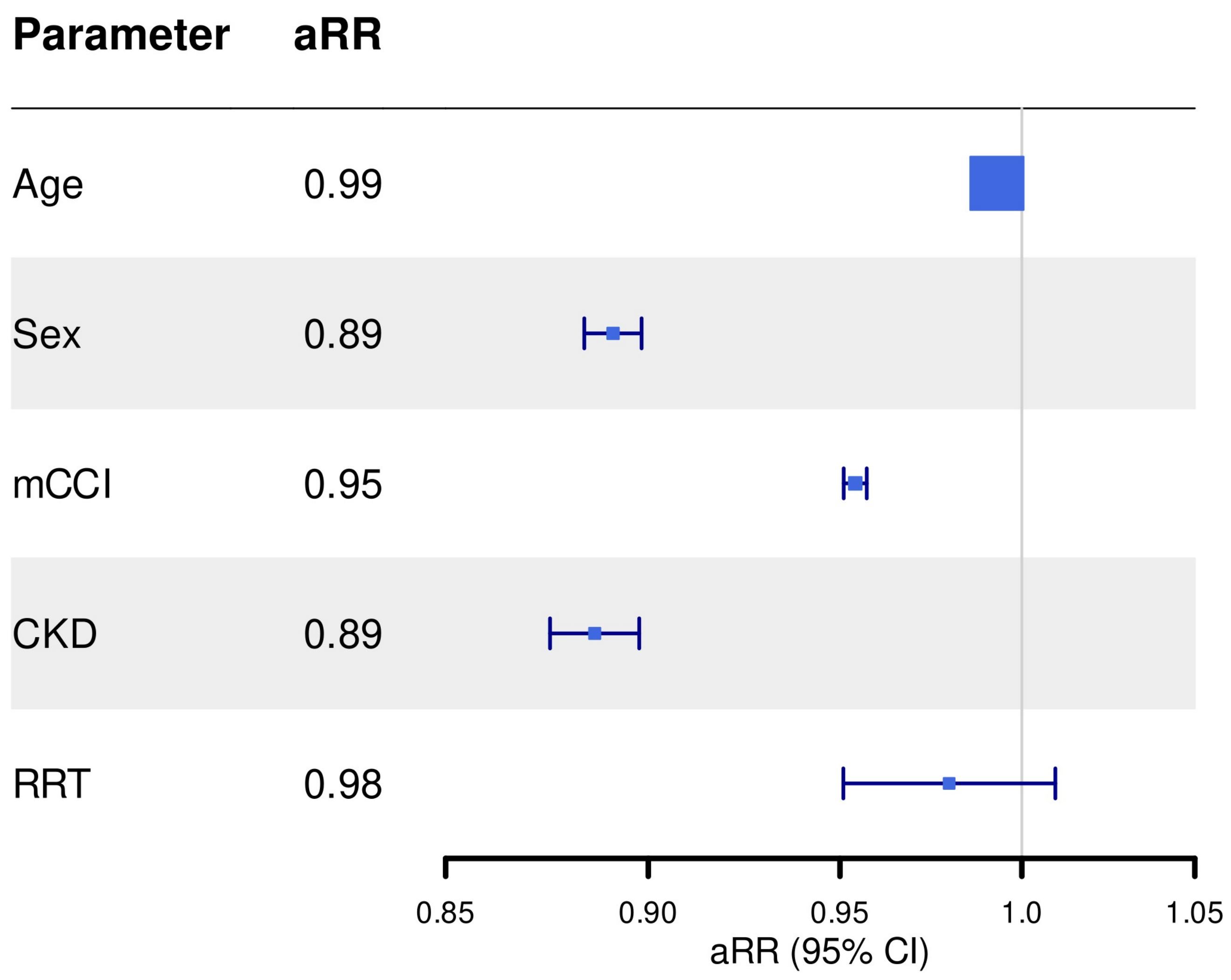

Supplement Fig. S4 PCI in STEMI in Germany. Plotted regression coefficients and 95% confidence Intervals.
