# Supplementary material for: Management of acute myocardial infarction in chronic kidney disease in Germany: an observational study
Source: BMC Nephrol. 2025 Jan 9;26:15. doi: 10.1186/s12882-025-03943-5 (PMC11720599; doi:10.1186/s12882-025-03943-5)
Supplement: Supplementary file 6 — Supplementary Material 6 [file 12882_2025_3943_MOESM6_ESM.pdf]

Supplement Table S1 ICD-10-GM- and OPS-Codes

**ICD-10 Code**	**Description**
**Chronic kidney disease**	
2016	
N18.3	Chronic kidney disease, stage 3
N18.4	Chronic kidney disease, stage 4
N18.5	Chronic kidney disease, stage 5
**Myocardial infarction**	
**STEMI**	
I21.0	Acute transmural myocardial infarction of the anterior wall
I21.1	Acute transmural myocardial infarction of the posterior wall
I21.2	Acute transmural myocardial infarction at other sites
I21.3	Acute transmural myocardial infarction of unspecified location
**NSTEMI**	
I21.4	Acute subendocardial myocardial infarction
I21.9	Acute myocardial infarction, unspecified
**Z49.- Dialysis treatment**	
Z49.1	Extracorporeal dialysis
Z49.2	Other dialysis (incl. peritoneal dialysis)
**OPS Code**	
**Intermittent haemodialysis**	
**8-853 Hemofiltration**	
2016	
8-853.3	Intermittent, anticoagulation with heparin or without anticoagulation
8-853.4	Intermittent, anticoagulation with other substances
8-853.5	Prolonged intermittent, anticoagulation with heparin or without anticoagulation
8-853.6	Prolonged intermittent, anticoagulation with other substances
**8-854 Haemodialysis**	
8-854.2	Intermittent, anticoagulation with heparin or without anticoagulation
8-854.3	Intermittent, anticoagulation with other substances
8-854.4	Prolonged intermittent, anticoagulation with heparin or without anticoagulation
8-854.5	Prolonged intermittent, anticoagulation with other substances
**8-855 Hemodiafiltration**	
8-855.3	Intermittent, anticoagulation with heparin or without anticoagulation
8-855.4	Intermittent, anticoagulation with other substances

8-855.5	Prolonged intermittent, anticoagulation with heparin or without anticoagulation
8-855.6	Prolonged intermittent, anticoagulation with other substances
**Peritoneal dialysis**	
**8-857 Peritoneal dialysis**	
8-857.0	Intermittent, machine assisted (IPD)
8-857.1	Continuous, not machine assisted (CAPD)
8-857.2	Continuous, machine assisted (APD), with additional equipment.
8-857.x	Other
**PCI**	
**8-837 Percutaneous transluminal vascular intervention on heart and coronary vessels**	
2016	
8-837.0	Angioplasty (balloon)
8-837.k	Insertion of a non-drug-eluting stent
8-837.m	Insertion of a drug-eluting stent
8-837.p	Non-drug-eluting covered stent (stent-graft) insertion - aneurysm treatment
8-837.u	Insertion of a non-drug-eluting bifurcation stent
8-837.v	Insertion of a drug-eluting bifurcation stent
8-837.w	Insertion of a coated stent
8-83b.b	Type of balloons used
8-83b.b1	Antibody-coated balloons
8-83b.b6-9	A drug-eluting balloon on coronary vessels
**CABG**	
**5-361 Placement of an aortocoronary bypass**	
2016	
5-361	Placement of an aortocoronary bypass
5-362	Placement of aortocoronary bypass by minimally invasive technique
5-363	Other revascularization of the heart
8-851	Surgical external circulation (when heart-lung machine is used)
**Systemic thrombolysis**	
2016	
8-020.8	Systemic thrombolysis

Legend Supplement Table S1. STEMI: ST-Segment elevating myocardial infarction; NSTEMI: Non-ST-segment elevation myocardial infarction; CKD: Chronic kidney disease; RRT: Renal replacement therapy; PCI: Percutaneous coronary intervention; CABG: Coronary artery bypass graft; IPD:

intermittent peritoneal dialysis; CAPD: continuous ambulatory peritoneal dialysis; APD: automated peritoneal dialysis.
